# Is histamine intolerance a treatable subtype of fibromyalgia? evidence and clinical implications—narrative review

**DOI:** 10.3389/fpain.2026.1786437

**Published:** 2026-04-30

**Authors:** João Protásio Netto, José Fábio Lana, Alexandre Parma, Fábio Ramos Costa, Rubens Martins de Andrade, Mariana Garcia Martins Castro, Claudia Tambelli

**Affiliations:** 1 Federal University of Tocantins (UFT), Palmas, Brazil; 2 Brazilian Institute of Regenerative Medicine (BIRM), São Paulo, Brazil; 3 Palmas School of Public Health Foundation, Family and Community Medicine Program, Palmas, Brazil; 4 University of Campinas (UNICAMP), Campinas, Brazil

**Keywords:** central sensitization, chronic pain, diamine oxidase, fibromyalgia, histamine intolerance, mast cells, neuroinflammation, pain modulation

## Abstract

**Background:**

Fibromyalgia affects 2%–8% of the global population with suboptimal treatment outcomes. Emerging evidence suggests histamine intolerance, mediated by diamine oxidase (DAO) deficiency, may contribute to fibromyalgia pathophysiology in specific patient subgroups.

**Methods:**

We conducted a comprehensive narrative review of literature published 2000–2025, examining genetic polymorphisms, biochemical markers, and clinical studies investigating relationships between histamine intolerance, DAO deficiency, and fibromyalgia. Studies included genetic association analyses, randomized controlled trials, and population-based observational research.

**Results:**

Key findings revealed 74.5% prevalence of DAO deficiency-associated genetic variants in fibromyalgia patients vs. 66% in general population (*p* = 0.014), with cumulative variants correlating with increased symptom severity (∼7-point FIQ increase per allele). A randomized controlled trial (*n* = 100) demonstrated significant improvements with DAO supplementation: Pain Catastrophizing Scale decreased 8.4 points vs. 2.1 (placebo), and Fibromyalgia Impact Questionnaire improved 12.3 vs. 4.6 points (*p* < 0.003). Population studies showed strong associations with histamine-mediated conditions: 29% fibromyalgia prevalence among chronic pruritus patients and odds ratios of 1.30–2.50 for allergic comorbidities among 15,869 fibromyalgia patients.

**Conclusion:**

Current evidence supports systematic histamine intolerance assessment in fibromyalgia patients presenting with gastrointestinal symptoms, chronic pruritus, or treatment resistance. This review provides a practical clinical framework for identifying and managing this potentially treatable fibromyalgia subtype, though larger multicenter replication studies are needed to confirm findings and establish standardized diagnostic protocols.

## 1 Introduction

Fibromyalgia represents a paradigmatic example of the complexity inherent in chronic pain syndromes, affecting between 2% and 8% of the global population with a striking female predominance of approximately 9:1 (1, 2). The condition imposes substantial personal and societal burdens, with patients experiencing not only widespread musculoskeletal pain but also profound fatigue, cognitive dysfunction, sleep disturbances, and numerous somatic symptoms that significantly impair quality of life and functional capacity (3). Despite decades of research and the development of multiple therapeutic approaches, treatment outcomes remain disappointingly modest, with most interventions achieving response rates below 50% and many patients cycling through multiple medications without achieving adequate symptom control (4, 5).

The pathophysiology of fibromyalgia has evolved from early concepts of peripheral muscle pathology to current understanding emphasizing central nervous system dysfunction, particularly involving altered pain processing, central sensitization, and disrupted descending pain modulation (6, 7). However, the heterogeneity of symptom presentations and treatment responses strongly suggests that fibromyalgia may represent a collection of related conditions with distinct underlying mechanisms rather than a single homogeneous disorder. This heterogeneity has prompted increasing interest in identifying specific pathophysiological subtypes that might respond to targeted therapeutic interventions, moving toward a precision medicine approach in chronic pain management (8).

Recent years have witnessed growing attention to the potential role of histamine dysregulation in chronic pain conditions, with particular interest in the concept of histamine intolerance as a contributing factor to symptom generation and maintenance (9, 10). Histamine, a biogenic amine with pleiotropic physiological functions spanning immune responses, neurotransmission, gastric acid secretion, and vascular regulation, has long been recognized for its role in acute inflammatory responses and allergic reactions. However, emerging evidence suggests that chronic low-grade histamine excess, resulting from impaired degradation capacity, may contribute to diverse chronic symptoms including pain, gastrointestinal disturbances, headaches, and fatigue—symptoms that notably overlap with the fibromyalgia syndrome (11, 12).

The primary pathway for histamine degradation in the gastrointestinal tract involves diamine oxidase (DAO), an enzyme predominantly expressed in intestinal epithelial cells that serves as the first-line defense against dietary histamine (13). When DAO activity is insufficient, whether due to genetic polymorphisms, enzyme inhibition, or intestinal pathology, histamine can accumulate systemically and produce wide-ranging effects through its interaction with four distinct G-protein coupled receptors distributed throughout peripheral and central tissues (14). The clinical syndrome of histamine intolerance, while not universally accepted and lacking standardized diagnostic criteria, has gained recognition as a potential explanation for otherwise unexplained multisystem symptoms in susceptible individuals (15).

The theoretical basis for examining histamine intolerance in fibromyalgia rests on several converging lines of evidence and reasoning. First, the symptom profiles of the two conditions show remarkable overlap, encompassing not only pain but also gastrointestinal symptoms, headaches, fatigue, and cognitive difficulties (16). Second, mast cells—the primary tissue reservoirs of histamine—have been implicated in fibromyalgia pathophysiology through multiple studies demonstrating increased mast cell degranulation, elevated tryptase levels, and therapeutic responses to mast cell stabilizers in selected patients (17, 18). Third, histamine receptors are strategically positioned throughout pain processing pathways, from peripheral nociceptors to central pain structures, providing multiple potential sites for histamine-mediated pain modulation (19). Fourth, the demographic profile of histamine intolerance, predominantly affecting middle-aged women, aligns remarkably with the epidemiology of fibromyalgia (20).

This comprehensive review aims to synthesize and critically evaluate the emerging evidence linking histamine intolerance to fibromyalgia, with particular emphasis on potential mechanisms of pain generation and amplification. We examine genetic predisposition through DAO gene variants, biochemical evidence of enzyme deficiency, clinical associations with histamine-mediated conditions, and preliminary therapeutic evidence from recent clinical trials. Our goal is to provide clinicians and researchers with a thorough understanding of this potential relationship while maintaining appropriate scientific skepticism about preliminary findings. We also propose practical approaches for clinical assessment and management while acknowledging the significant knowledge gaps that require future research attention.

## 2 Materials and methods

### 2.1 Literature search strategy

We conducted a comprehensive literature review examining the relationship between histamine intolerance, diamine oxidase (DAO) deficiency, and fibromyalgia. A systematic search was performed across multiple electronic databases including PubMed/MEDLINE, Scopus, Web of Science, and Embase for publications from January 2000 to January 2025.

The search strategy employed a combination of medical subject headings (MeSH terms) and keywords including: “fibromyalgia” OR “chronic widespread pain” combined with “histamine intolerance” OR “diamine oxidase” OR “DAO deficiency” OR “DAO enzyme” OR “AOC1 gene” OR “histamine metabolism” OR “mast cell activation” OR “histamine receptors” OR “neuroinflammation” OR “biogenic amines.” Additional Boolean operators were used to refine searches and capture relevant literature. Reference lists of retrieved articles were manually screened to identify additional relevant publications not captured by the database searches.

### 2.2 Inclusion and exclusion criteria

Studies were included if they met the following criteria: (1) original research articles, clinical trials, genetic association studies, or population-based observational studies; (2) investigations examining histamine metabolism, DAO activity, or related mechanisms in fibromyalgia patients or relevant animal models; (3) studies assessing clinical associations between histamine-mediated conditions and fibromyalgia; (4) therapeutic trials evaluating histamine-targeted interventions in fibromyalgia; and (5) publications in English, Spanish, or Portuguese.

Exclusion criteria included: (1) case reports or case series with fewer than 10 patients; (2) conference abstracts without full-text availability; (3) studies lacking clear methodology or appropriate controls; and (4) publications focusing solely on other chronic pain conditions without fibromyalgia-specific data.

Both human studies and relevant preclinical investigations were considered when they provided mechanistic insights into histamine's role in pain processing, neuroinflammation, or central sensitization applicable to fibromyalgia pathophysiology.

### 2.3 Data extraction and quality assessment

Data were systematically extracted from included studies using a standardized form capturing: (1) study characteristics (author, year, country, design, sample size); (2) population characteristics (demographics, diagnostic criteria, disease duration); (3) genetic data (AOC1 variants, allele frequencies, genotype distributions); (4) biochemical markers (DAO activity levels, histamine metabolites); (5) clinical outcomes (symptom scores, quality of life measures, treatment responses); and (6) key findings and limitations.

For genetic studies, we extracted minor allele frequencies, odds ratios, and gene-dose effects. For clinical trials, we recorded intervention details, primary and secondary outcomes, effect sizes, and adverse events. For observational studies, we captured prevalence rates, odds ratios, and adjusted analyses.

Study quality was evaluated based on: sample size adequacy, appropriate control groups, validated outcome measures, clear diagnostic criteria, adequate follow-up duration, and transparent reporting of potential biases and limitations. Randomized controlled trials were assessed using Cochrane Risk of Bias tools, while observational studies were evaluated for selection bias, confounding, and measurement accuracy.

### 2.4 Data synthesis and analysis

Given the heterogeneity of study designs, populations, and outcome measures, a narrative synthesis approach was employed rather than formal meta-analysis. Evidence was organized thematically into: (1) genetic and biochemical evidence; (2) clinical phenotypes and associations; (3) mechanistic pathways; (4) therapeutic interventions; and (5) clinical implementation strategies.

Findings were critically evaluated considering methodological quality, consistency across studies, biological plausibility, and clinical relevance. Where multiple studies addressed similar questions, we assessed concordance of findings and explored potential sources of heterogeneity. Limitations and knowledge gaps were systematically identified to guide future research priorities.

The strength of evidence was characterized qualitatively as: strong (consistent findings across multiple high-quality studies), moderate (some supporting evidence with limitations), preliminary (limited studies or significant methodological constraints), or insufficient (conflicting or very limited data). This framework informed conclusions and clinical recommendations while maintaining appropriate scientific caution about preliminary findings.

## 3 Histamine metabolism, DAO function, and pain pathways

### 3.1 The biochemistry and physiology of histamine metabolism

Understanding the potential role of histamine in fibromyalgia requires detailed knowledge of histamine metabolism and the consequences of metabolic dysfunction. Histamine (2-[4-imidazolyl]ethylamine) is synthesized from the amino acid L-histidine through the action of histidine decarboxylase, an enzyme expressed in multiple cell types including mast cells, basophils, enterochromaffin-like cells, and specific neuronal populations (21). Once synthesized, histamine is either stored in secretory granules for regulated release or immediately metabolized, depending on the cellular context and physiological requirements.

The degradation of histamine occurs through two primary enzymatic pathways with distinct tissue distributions and physiological roles. Diamine oxidase (DAO), also known as amiloride-binding protein 1 or histaminase, represents the primary barrier preventing dietary histamine from entering systemic circulation (22). This copper-containing amine oxidase is predominantly expressed in the intestinal epithelium, particularly in villus tips of the small intestine, but also in the kidney, placenta, and to a lesser extent in other tissues. DAO functions as a secreted enzyme, released from intestinal cells into the gut lumen and circulation, where it catalyzes the oxidative deamination of histamine to imidazole acetaldehyde, which is subsequently converted to imidazole acetic acid and its riboside conjugate (23).

**Figure 1:**
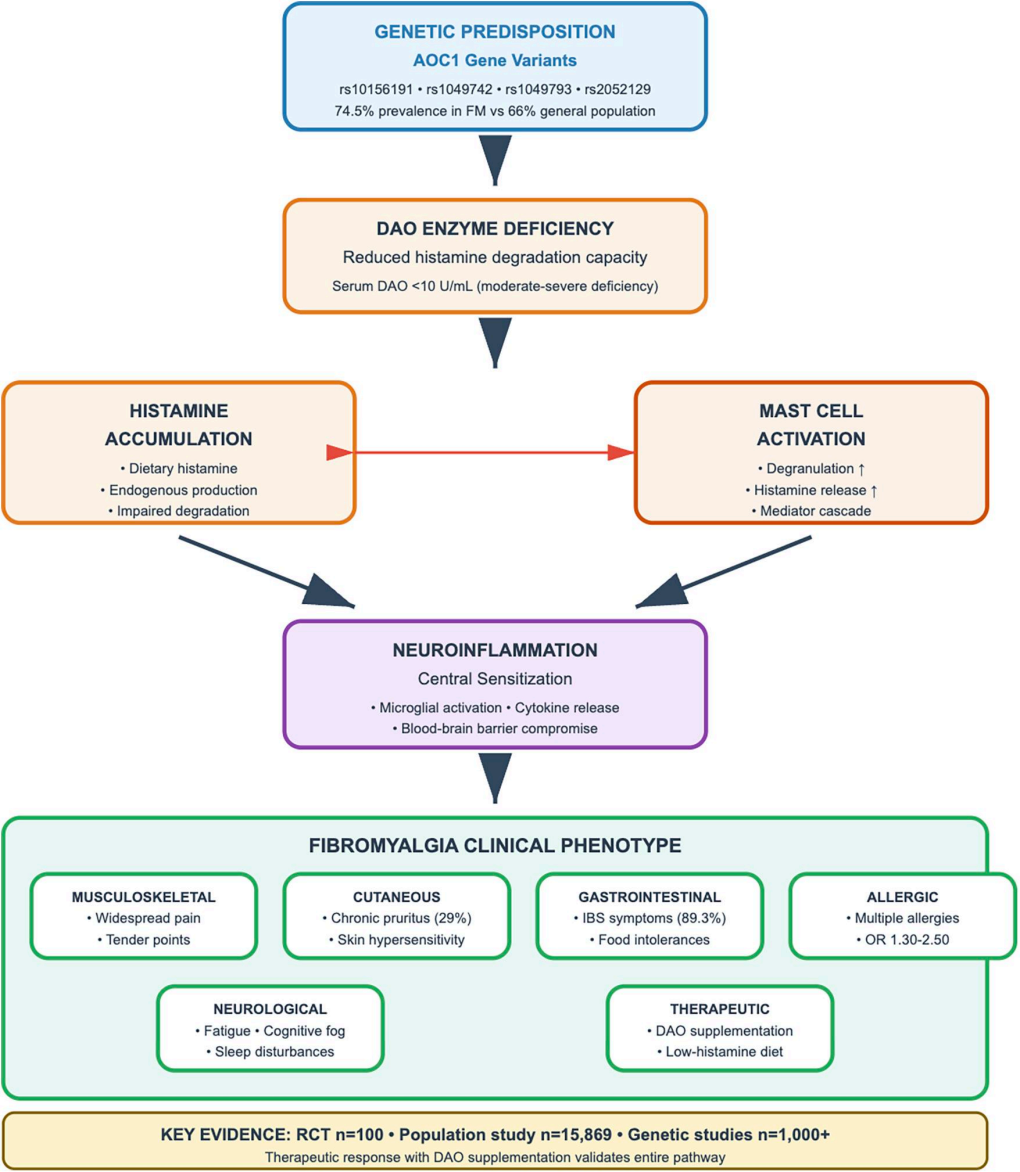
Histamine-Fibromyalgia pathophysiological axis. This schematic illustrates the cascade from genetic predisposition (AOC1 gene variants) through DAO enzyme deficiency, histamine accumulation, mast cell activation, and neuroinflammation, ultimately leading to the diverse fibromyalgia clinical phenotype including musculoskeletal, cutaneous, gastrointestinal, allergic, and neurological manifestations. The figure demonstrates therapeutic intervention points including DAO supplementation and low-histamine diet. Key evidence supporting the pathway includes RCT data (*n* = 100), population studies (*n* = 15,869), and genetic validation studies (*n* = 1,000+).

The second major pathway involves histamine N-methyltransferase (HNMT), a cytosolic enzyme that catalyzes the methylation of histamine to N-methylhistamine, which is further oxidized by monoamine oxidase B to N-methylimidazole acetic acid (24). HNMT shows highest expression in the liver and kidney but is also present in the brain, where it represents the primary mechanism for terminating histaminergic neurotransmission. The relative contributions of these two pathways vary by tissue and physiological context, with DAO being critical for managing exogenous (dietary) histamine and HNMT primarily handling endogenous histamine, particularly in the central nervous system (25). Figure 1 illustrates this complete pathophysiological cascade from genetic variants through enzymatic deficiency to clinical manifestations.

The AOC1 gene encoding DAO, located on chromosome 7q34-q36, spans approximately 10 kilobases and contains five exons (26). Genetic variations in AOC1 can significantly impact enzyme expression and activity, with four single nucleotide polymorphisms (SNPs) receiving particular attention for their functional consequences. The rs10156191 (c.47T>C) variant in the promoter region affects transcriptional efficiency, while rs1049742 (c.995C>T) and rs1049793 (c.1990C>G) result in amino acid substitutions that alter enzyme stability and catalytic activity. The rs2052129 (c.691G>T) variant, causing a valine to phenylalanine substitution, has been associated with reduced enzyme secretion (27, 28). Population genetics studies indicate considerable variation in the prevalence of these variants across ethnic groups, with compound heterozygosity and homozygosity for multiple variants showing cumulative effects on enzyme function (29).

Beyond genetic factors, DAO activity can be modulated by numerous environmental and physiological influences. Alcohol consumption, particularly red wine and beer rich in histamine and other biogenic amines, can competitively inhibit DAO while simultaneously providing substrate load (30). Various medications including nonsteroidal anti-inflammatory drugs, certain antibiotics, and antidepressants have been reported to inhibit DAO activity, potentially precipitating histamine intolerance symptoms in susceptible individuals (31). Gastrointestinal conditions affecting intestinal integrity, such as inflammatory bowel disease, celiac disease, and small intestinal bacterial overgrowth, can reduce DAO expression and release, creating acquired deficiency states (32). Additionally, DAO activity shows developmental regulation, being low in infancy and increasing with age, and hormonal influences, with pregnancy dramatically increasing DAO production by the placenta to protect the developing fetus from maternal histamine (33).

### 3.2 Histamine receptors and pain signaling mechanisms

The biological effects of histamine are mediated through four distinct G-protein coupled receptors (H1-H4), each with unique tissue distributions, signaling mechanisms, and physiological functions relevant to pain processing (34). Understanding these receptor systems is crucial for comprehending how histamine dysregulation might contribute to fibromyalgia symptoms and for identifying potential therapeutic targets.

The H1 receptor, coupled to Gq/11 proteins, activates phospholipase C leading to inositol triphosphate production and intracellular calcium mobilization (35). In the context of pain, H1 receptors on primary sensory neurons mediate the classic inflammatory response, including vasodilation, increased vascular permeability, and direct nociceptor activation. Peripheral H1 receptor activation lowers nociceptor thresholds and increases responsiveness to other algesic mediators, contributing to peripheral sensitization—a key mechanism in chronic pain states (36). In the central nervous system, H1 receptors are widely distributed throughout pain processing regions including the thalamus, somatosensory cortex, and periaqueductal gray, where they modulate synaptic transmission and neuronal excitability. The sedating effects of first-generation H1 antagonists highlight the importance of central H1 receptors in arousal and sleep-wake regulation, functions notably disrupted in fibromyalgia (37).

H2 receptors, primarily coupled to Gs proteins and adenylyl cyclase activation, have received less attention in pain research but may play important roles in both peripheral and central pain processing (38). While best known for regulating gastric acid secretion, H2 receptors on immune cells modulate cytokine production and inflammatory responses. In the nervous system, H2 receptors can enhance long-term potentiation and synaptic plasticity, processes implicated in pain chronification. The high prevalence of gastrointestinal symptoms in fibromyalgia raises questions about potential H2 receptor involvement in visceral hypersensitivity and gut-brain axis dysfunction (39).

The H3 receptor functions primarily as a presynaptic autoreceptor and heteroreceptor, regulating the release of histamine and other neurotransmitters including acetylcholine, dopamine, norepinephrine, and serotonin (40). This regulatory role positions H3 receptors as potential modulators of descending pain control systems, which show documented dysfunction in fibromyalgia. H3 receptor inverse agonists have shown analgesic properties in preclinical models, suggesting that enhanced histaminergic tone through H3 receptor modulation might paradoxically reduce pain under certain conditions. The complex bidirectional effects of histamine on pain—both pronociceptive and antinociceptive depending on receptor subtype and location—highlight the importance of understanding receptor-specific actions (41).

The most recently discovered H4 receptor, predominantly expressed on immune cells including mast cells, eosinophils, and T lymphocytes, has emerged as a key player in inflammatory and neuropathic pain (42). H4 receptor activation promotes chemotaxis and inflammatory mediator release, contributing to neuroinflammation—a process increasingly recognized in fibromyalgia pathophysiology. Preclinical studies demonstrate that H4 receptor antagonists reduce mechanical and thermal hyperalgesia in various pain models, suggesting therapeutic potential. The presence of H4 receptors on mast cells creates potential positive feedback loops where histamine release triggers further mast cell activation and degranulation (43).

### 3.3 Neuroinflammation and the histamine-mast cell-microglia axis

The concept of neuroinflammation has revolutionized understanding of chronic pain conditions, including fibromyalgia, shifting focus from purely neuronal mechanisms to the complex interplay between neurons, glia, and immune cells (44). Within this framework, the histamine-mast cell-microglia axis emerges as a potentially critical pathway linking peripheral immune activation to central sensitization and sustained pain states.

Mast cells, strategically positioned at the interface between the nervous and immune systems, serve as sentinel cells capable of rapidly releasing diverse mediators including histamine, tryptase, cytokines, and chemokines (45). In fibromyalgia, multiple studies have documented increased mast cell degranulation in skin biopsies, elevated serum tryptase levels, and therapeutic responses to mast cell stabilizers in selected patients, suggesting mast cell involvement in symptom generation (46, 47). The proximity of mast cells to sensory nerve endings enables bidirectional communication through which neuropeptides like substance P and calcitonin gene-related peptide trigger mast cell degranulation, while mast cell mediators sensitize nociceptors—a process termed neurogenic inflammation (48).

Within the central nervous system, resident mast cells, though less numerous than in peripheral tissues, occupy strategic locations including the thalamus, hypothalamus, and dural matter, where they can influence pain processing, neuroendocrine function, and headache generation (49). Recent evidence suggests that stress, a well-recognized fibromyalgia trigger, can activate CNS mast cells through corticotropin-releasing hormone receptors, leading to increased blood-brain barrier permeability and facilitating peripheral-to-central inflammatory signaling (50, 51). This mechanism could explain how peripheral triggers translate into central symptoms and why stress management remains crucial in fibromyalgia treatment (52).

Microglia, the resident immune cells of the central nervous system, represent another critical component of neuroinflammation in chronic pain (53). Histamine, whether derived from peripheral sources crossing a compromised blood-brain barrier or from central mast cells, can directly activate microglia through H1 and H4 receptors. Activated microglia release pro-inflammatory cytokines (TNF-α, IL-1β, IL-6), chemokines, and additional histamine, creating self-perpetuating inflammatory cascades (54). Microglial activation has been visualized in fibromyalgia patients using positron emission tomography, providing direct evidence for central neuroinflammation. The temporal dynamics of microglial activation, with potential transitions from acute protective responses to chronic pathological states, may explain the evolution from acute to chronic pain in susceptible individuals (55).

The interaction between histamine and other pain-relevant neurotransmitter systems adds another layer of complexity to fibromyalgia pathophysiology. Histamine modulates serotonergic neurotransmission, potentially contributing to the mood disturbances and altered pain modulation observed in fibromyalgia (56). The well-documented efficacy of serotonin-norepinephrine reuptake inhibitors in fibromyalgia might partially reflect normalization of histamine-serotonin interactions. Similarly, histamine influences dopaminergic systems involved in pain perception and reward processing, potentially contributing to the anhedonia and altered pain-pleasure balance reported by many fibromyalgia patients (57).

## 4 Results: clinical evidence linking histamine intolerance to fibromyalgia

### 4.1 Genetic epidemiology and DAO variants in fibromyalgia populations

**Figure 2:**
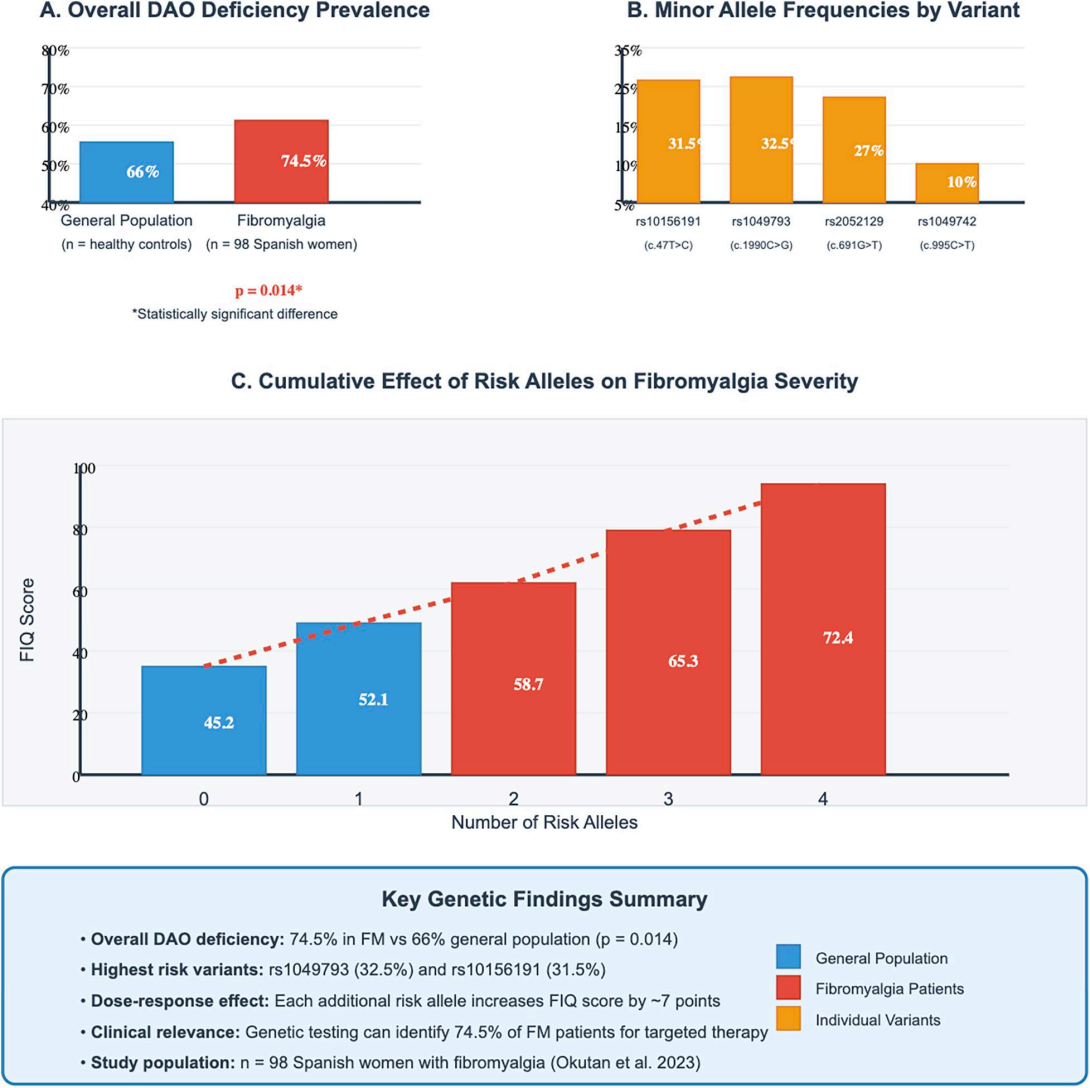
Genetic evidence for DAO deficiency in fibromyalgia - AOC1 gene variants and population comparisons. **(A)** Shows overall DAO deficiency prevalence comparing fibromyalgia patients (74.5%, *n* = 98) vs. general population (66%, *n* = 308 newborns), with statistical significance (*p* = 0.014). **(B)** Displays minor allele frequencies for the four major variants: rs10156191 (31.5%), rs1049793 (32.5%), rs2052129 (27%), and rs1049742 (10%). **(C)** Demonstrates the cumulative effect of risk alleles on fibromyalgia severity (FIQ scores), showing dose-dependent relationship: 0 alleles (45.2), 1 allele (52.1), 2 alleles (58.7), 3 alleles (65.3), and 4 alleles (72.4). Key findings summary box indicates 74.5% overall DAO deficiency, highest risk variants, ∼7-point FIQ increase per allele, and clinical relevance for identifying 74.5% of FM patients for targeted therapy.

The genetic architecture of DAO deficiency in fibromyalgia populations has been illuminated by recent studies examining AOC1 gene variants and their functional consequences. The landmark work by Okutan and colleagues (2023) represents the most comprehensive genetic analysis to date, examining four major SNPs in 98 Spanish women with fibromyalgia diagnosed according to American College of Rheumatology 2010 criteria (Supplementary Table S1) (58). Their findings revealed that 74.5% of fibromyalgia patients carried at least one variant allele associated with reduced DAO activity, compared to the 66% prevalence reported in the general Spanish population—a statistically significant difference (*p* = 0.014) that suggests genetic enrichment for DAO deficiency in fibromyalgia (Figure 2).

The distribution of individual variants provides insights into potential functional impacts. The rs10156191 variant, located in the promoter region and affecting transcriptional regulation, showed a minor allele frequency of 31.5% in fibromyalgia patients. The rs1049793 variant, causing a histidine to glutamine substitution at position 664, demonstrated similar prevalence at 32.5%. The rs2052129 variant, resulting in a valine to phenylalanine substitution at position 231 that affects enzyme secretion, occurred in 27% of patients. The rs1049742 variant, while less common at 10%, causes a threonine to isoleucine change at position 332 that significantly reduces catalytic activity (see Supplementary Table S2 for complete variant details) (59).

Perhaps most compelling was the demonstration of a gene-dose effect, with cumulative risk alleles correlating with fibromyalgia severity as measured by the Fibromyalgia Impact Questionnaire (FIQ). Patients carrying no risk alleles had mean FIQ scores of 45.2, while those with one, two, three, and four risk alleles showed progressively higher scores of 52.1, 58.7, 65.3, and 72.4, respectively. This approximately 7-point increase per additional risk allele suggests that genetic burden translates into clinical severity, supporting a potential causal relationship rather than mere association (60).

The prevalence data gain additional context from population genetics studies. Fortes Marin and colleagues (2025) examined 308 healthy Spanish newborns, finding a 66% prevalence of at least one DAO deficiency-associated variant, establishing baseline population frequencies (61). Interestingly, studies in other clinical populations have revealed varying prevalence rates: 78.8% in attention-deficit/hyperactivity disorder, 82.6% in insomnia-related symptoms, and 79%–83% in patients with diagnosed histamine intolerance symptoms. These differential prevalence rates across conditions suggest that DAO deficiency might represent a shared vulnerability factor for multiple disorders characterized by central nervous system dysfunction and systemic symptoms (62).

The functional consequences of these genetic variants extend beyond simple enzyme deficiency. Recent molecular studies indicate that certain variant combinations affect not only enzyme activity but also cellular trafficking, post-translational modifications, and response to cofactors like copper and vitamin B6 (63). This complexity suggests that genetic testing alone may not fully capture functional DAO status, highlighting the need for integrated genetic-biochemical assessment approaches. Additionally, epigenetic factors, including promoter methylation and histone modifications, may modulate AOC1 expression independently of genetic variants, potentially explaining some of the variability in clinical presentations among genetically similar individuals.

### 4.2 Biochemical markers and functional assessment

The translation from genetic predisposition to functional enzyme deficiency represents a critical step in understanding the histamine-fibromyalgia relationship. Serum DAO activity, while not standardized across laboratories, provides a functional measure that integrates genetic, epigenetic, and environmental influences on enzyme availability and activity. Established clinical thresholds, though requiring validation in larger populations, suggest that DAO activity below 3 U/mL indicates severe deficiency likely to produce symptoms, 3–10 U/mL represents moderate deficiency with variable clinical manifestations, and above 10 U/mL is considered normal, though this may not exclude localized intestinal deficiency (64).

Studies examining DAO levels in fibromyalgia have consistently reported reduced enzyme activity compared to healthy controls, though absolute values vary depending on methodology (65). The correlation between genetic variants and functional enzyme activity strengthens the biological plausibility of DAO deficiency as a contributing factor. However, the relationship is not deterministic—some individuals with high-risk genotypes maintain normal enzyme activity, while others without genetic variants show functional deficiency, highlighting the importance of comprehensive assessment (66).

Beyond static DAO measurements, dynamic testing approaches have been proposed to better capture functional histamine intolerance. Histamine provocation tests, while controversial due to safety concerns and lack of standardization, have shown altered responses in fibromyalgia patients, including exaggerated symptom reproduction and delayed histamine clearance (67). The measurement of histamine metabolites, particularly N-methylhistamine in urine, provides an indirect assessment of histamine turnover and has shown elevation in subsets of fibromyalgia patients, suggesting increased histamine production or decreased degradation. The ratio of histamine to its metabolites may provide a more comprehensive picture of histamine metabolism than isolated DAO measurements (68).

### 4.3 Clinical phenotypes and symptom associations

**Figure 3:**
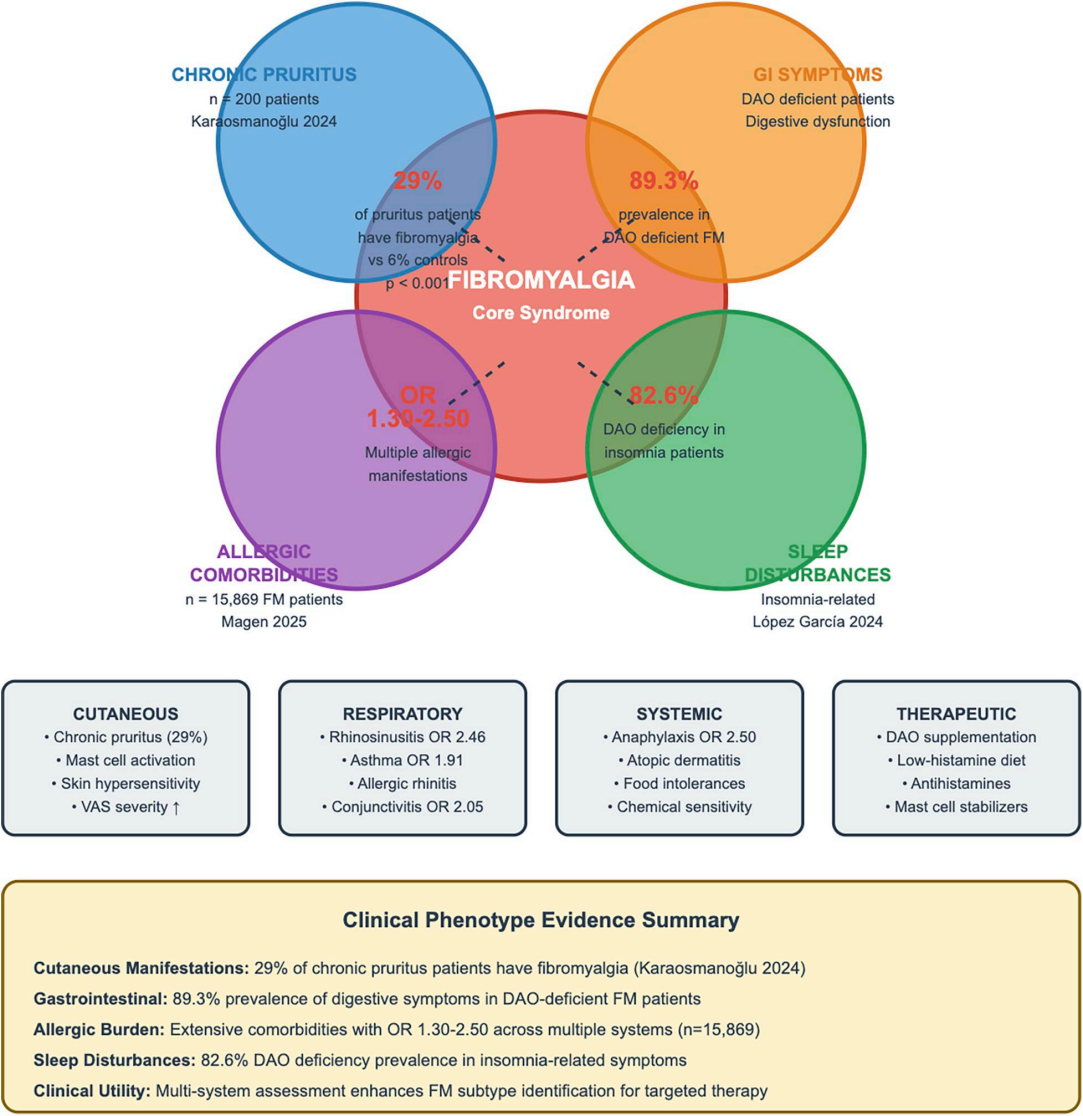
Expanded fibromyalgia clinical phenotype - histamine-mediated manifestations and comorbidities. This Venn diagram illustrates the overlapping manifestations of fibromyalgia and histamine-related conditions. The central core represents fibromyalgia syndrome, with four major domains overlapping: Chronic Pruritus (29% of pruritus patients have fibromyalgia, Karaosmanoğlu 2024), GI Symptoms (89.3% prevalence in DAO deficient FM patients), Sleep Disturbances (82.6% DAO deficiency in insomnia patients, López García 2024), and Allergic Comorbidities (OR 1.30-2.50 across multiple manifestations, *n* = 15,869, Magen 2025). Bottom panels detail specific manifestations: Cutaneous (chronic pruritus 29%, mast cell activation, VAS severity), Respiratory (rhinosinusitis OR 2.46, asthma OR 1.91), Systemic (anaphylaxis OR 2.50, food intolerances), and Therapeutic approaches (DAO supplementation, low-histamine diet, antihistamines, mast cell stabilizers). Clinical Phenotype Evidence Summary box emphasizes multi-system assessment for FM subtype identification.

The clinical manifestations potentially linking histamine intolerance to fibromyalgia extend beyond pain to encompass multiple organ systems, reflecting histamine's pleiotropic physiological roles (Figure 3). Understanding these associations helps identify patients most likely to benefit from histamine-targeted interventions and provides insights into shared pathophysiological mechanisms.

#### 4.3.1 Gastrointestinal manifestations

The gastrointestinal tract, as the primary site of DAO expression and dietary histamine exposure, unsurprisingly shows prominent involvement in fibromyalgia patients with suspected histamine intolerance. Studies report that up to 89.3% of fibromyalgia patients with documented DAO deficiency experience significant gastrointestinal symptoms, including bloating, abdominal pain, altered bowel habits, and food intolerances (69). The overlap with irritable bowel syndrome (IBS), present in 40%–80% of fibromyalgia patients, raises questions about shared mechanisms. Histamine's effects on intestinal motility, secretion, and visceral sensitivity could contribute to both conditions. The improvement in gastrointestinal symptoms following low-histamine diets or DAO supplementation in fibromyalgia patients provides functional validation of this connection (70).

#### 4.3.2 Cutaneous and allergic manifestations

The striking finding by Karaosmanoğlu and colleagues (2024) that 29% of patients with chronic pruritus have concomitant fibromyalgia, compared to only 6% in controls, provides compelling evidence for shared histamine-mediated mechanisms (71). The severity correlation—with 55.2% of fibromyalgia patients experiencing very severe pruritus vs. 35.2% without fibromyalgia—suggests that fibromyalgia may amplify histamine-mediated symptoms. The median pruritus duration of 32 months parallels the chronic course of fibromyalgia, supporting sustained rather than episodic histamine dysregulation.

The comprehensive population-based study by Magen and colleagues (2025), analyzing 15,869 fibromyalgia patients over a 21-year period, revealed extensive allergic comorbidities with impressive odds ratios: anaphylactic reactions (OR 2.50), chronic rhinosinusitis (OR 2.46), allergic conjunctivitis (OR 2.05), bronchial asthma (OR 1.91), and atopic dermatitis (OR 1.41) (72). These associations remained significant after adjusting for confounders, suggesting intrinsic relationships rather than coincidental comorbidity. The breadth of allergic manifestations—spanning respiratory, cutaneous, and systemic reactions—indicates systemic histamine dysregulation rather than organ-specific pathology.

#### 4.3.3 Neurological and cognitive symptoms

Histamine's role as a neurotransmitter positions it to influence the neurological symptoms prominent in fibromyalgia. The association between DAO deficiency and sleep disturbances, with 82.6% of patients with insomnia-related symptoms showing genetic DAO deficiency, aligns with histamine's established role in sleep-wake regulation (73). Fibromyalgia patients frequently report non-restorative sleep, and histamine excess could contribute through multiple mechanisms: direct arousal promotion via H1 receptors, disruption of sleep architecture, and inflammatory mediator release affecting sleep quality.

The cognitive symptoms termed “fibro fog"—including attention deficits, memory impairment, and executive dysfunction—may partially reflect histamine dysregulation. Histamine modulates attention and learning through H3 receptors, and the 78.8% prevalence of DAO deficiency in ADHD patients suggests potential cognitive consequences of histamine excess (74). The improvement in cognitive symptoms reported by some fibromyalgia patients following antihistamine treatment, while anecdotal, warrants systematic investigation. A comprehensive summary of clinical features associated with histamine intolerance in fibromyalgia, including prevalence rates and supporting evidence for gastrointestinal, cutaneous, neurological, and allergic manifestations, is provided in Supplementary Table S3.

### 4.4 Therapeutic evidence from clinical trials

The most direct evidence for the histamine-fibromyalgia connection comes from interventional studies targeting histamine metabolism. The randomized, double-blind, placebo-controlled trial by Okutan and colleagues (2023) represents a landmark in this field, providing the first Level I evidence for therapeutic benefit from addressing histamine intolerance in fibromyalgia (75).

This trial enrolled 100 women with fibromyalgia (ages 33–61 years, mean 47.3 ± 8.2) who met ACR 2010 criteria and had symptom duration exceeding one year. Participants were randomized to receive either DAO supplementation (commercially available pig kidney-derived DAO, 4.2 mg taken 20 min before main meals) or identical placebo for 8 weeks. The primary outcome was change in Pain Catastrophizing Scale (PCS) scores, with secondary outcomes including Fibromyalgia Impact Questionnaire, fatigue visual analog scales, and gastrointestinal symptom ratings.

Results showed statistically significant improvements in the DAO group compared to placebo across multiple domains. The PCS total score decreased by 8.4 points in the DAO group vs. 2.1 points in placebo (*p* < 0.001), with particular improvements in rumination and helplessness subscales. FIQ scores improved by 12.3 points vs. 4.6 points (*p* = 0.003), exceeding the minimal clinically important difference. Notably, gastrointestinal symptoms showed marked improvement, with 68% of DAO-treated patients reporting reduced bloating and abdominal discomfort vs. 24% in the placebo group. The treatment was well-tolerated with no significant adverse events, though mild initial gastrointestinal symptoms occurred in 12% of the DAO group (76).

While these results are encouraging, several limitations warrant consideration. The 8-week duration, while sufficient to demonstrate initial efficacy, doesn't address long-term effectiveness or safety. The exclusion of men and narrow age range limits generalizability. The lack of baseline DAO measurement or genetic stratification represents a missed opportunity to identify optimal responders. Additionally, the use of pig-derived DAO raises questions about antigenicity with long-term use and the need for plant-based or recombinant alternatives.

Complementary evidence comes from dietary intervention studies. Gómez-Argüelles and colleagues (2022) examined 84 fibromyalgia patients following histamine-restricted diets based on specific histamine release testing, demonstrating significant improvements in both gastrointestinal and systemic symptoms compared to controls following standard dietary advice (77). The improvement correlated with dietary compliance, supporting a dose-response relationship. However, the complexity of dietary interventions, potential placebo effects, and difficulty maintaining long-term adherence complicate interpretation.

## 5 Discussion: mechanistic integration and clinical implications

### 5.1 Peripheral mechanisms of histamine-mediated pain amplification

The contribution of histamine to fibromyalgia pain likely begins at the peripheral level, where multiple mechanisms converge to amplify nociceptive signaling. Histamine released from mast cells, whether triggered by mechanical stimuli, stress mediators, or inflammatory signals, directly activates and sensitizes polymodal nociceptors through H1 and H4 receptors (78). This activation lowers mechanical and thermal thresholds, increases spontaneous discharge, and enhances responses to other algesic mediators—hallmarks of peripheral sensitization observed in fibromyalgia.

The concept of neurogenic inflammation provides a framework for understanding how initial nociceptor activation creates self-perpetuating cycles of sensitization. Antidromic activation of sensitized C-fibers releases neuropeptides including substance P and CGRP, which trigger further mast cell degranulation and histamine release. This positive feedback loop could explain the spreading and persistence of pain in fibromyalgia, where initial local symptoms evolve into widespread pain. The increased skin mast cell density and degranulation observed in fibromyalgia patients provides anatomical substrate for enhanced neurogenic inflammation (79).

Histamine's vascular effects compound peripheral sensitization through multiple mechanisms. H1-mediated vasodilation and increased vascular permeability facilitate inflammatory cell infiltration and mediator accumulation. The resulting tissue edema increases mechanical pressure on nociceptors while impairing metabolite clearance. Fibromyalgia patients show altered microcirculation and reduced capillary density, potentially reflecting chronic histamine-mediated vascular changes. The improvement in peripheral blood flow following antihistamine treatment in some patients supports vascular involvement (80).

### 5.2 Central mechanisms and neuroinflammation

The transition from peripheral to central sensitization represents a critical step in fibromyalgia development, and histamine appears positioned to facilitate this progression through multiple pathways. Within the spinal cord, histamine can enhance synaptic transmission between primary afferents and second-order neurons through presynaptic facilitation and postsynaptic depolarization. H1 receptors on dorsal horn neurons increase excitability and reduce inhibitory tone, effectively amplifying ascending pain signals. The presence of mast cells in the spinal meninges provides a local source of histamine that could sustain central sensitization (81).

The blood-brain barrier, traditionally viewed as limiting peripheral histamine access to the CNS, shows increased permeability in chronic pain states including fibromyalgia (80, 82). Stress-induced mast cell activation, peripheral inflammation, and sleep deprivation—all common in fibromyalgia—compromise barrier integrity, potentially allowing peripheral histamine and inflammatory mediators to access central pain processing regions (83, 84). This mechanism could explain why peripheral triggers produce central symptoms and why systemic anti-inflammatory interventions sometimes provide benefit (85).

Within the brain, histaminergic neurons originating in the tuberomammillary nucleus project widely to pain-relevant regions including the thalamus, somatosensory cortex, anterior cingulate cortex, and periaqueductal gray. Dysregulation of this histaminergic system could contribute to multiple fibromyalgia symptoms: altered pain perception through thalamic and cortical effects, emotional aspects of pain through limbic projections, and sleep disturbances through arousal system modulation. The high density of H3 autoreceptors in these regions suggests potential for feedback dysregulation maintaining altered histaminergic tone (30).

### 5.3 The stress-histamine-pain axis

Stress represents one of the most consistent fibromyalgia triggers, and the bidirectional relationship between stress and histamine provides mechanistic insights into this clinical observation. Acute stress activates mast cells through both direct neural pathways (sympathetic innervation) and hormonal mechanisms (corticotropin-releasing hormone), leading to histamine release. Chronic stress induces mast cell proliferation and reduces DAO expression, creating a state of increased histamine production and decreased degradation capacity (86).

The hypothalamic-pituitary-adrenal (HPA) axis, showing documented dysfunction in fibromyalgia, interfaces with histamine systems at multiple levels. Histamine stimulates ACTH release, potentially contributing to the altered cortisol rhythms observed in fibromyalgia. Conversely, glucocorticoids modulate mast cell function and DAO expression, creating complex feedback loops. The blunted cortisol response to stress in many fibromyalgia patients might impair normal histamine regulation, allowing excessive or prolonged histamine effects (87).

Psychological stress also influences pain perception through histamine-dependent mechanisms. Anxiety, common in fibromyalgia, is associated with increased mast cell activation and histamine release in brain regions processing threat and pain (88, 89). The anxiolytic effects of certain antihistamines suggest histamine involvement in anxiety generation. The improvement in both pain and anxiety with stress management interventions might partially reflect normalization of histamine dynamics (90).

### 5.4 Synthesis and evolution of evidence

**Figure 4:**
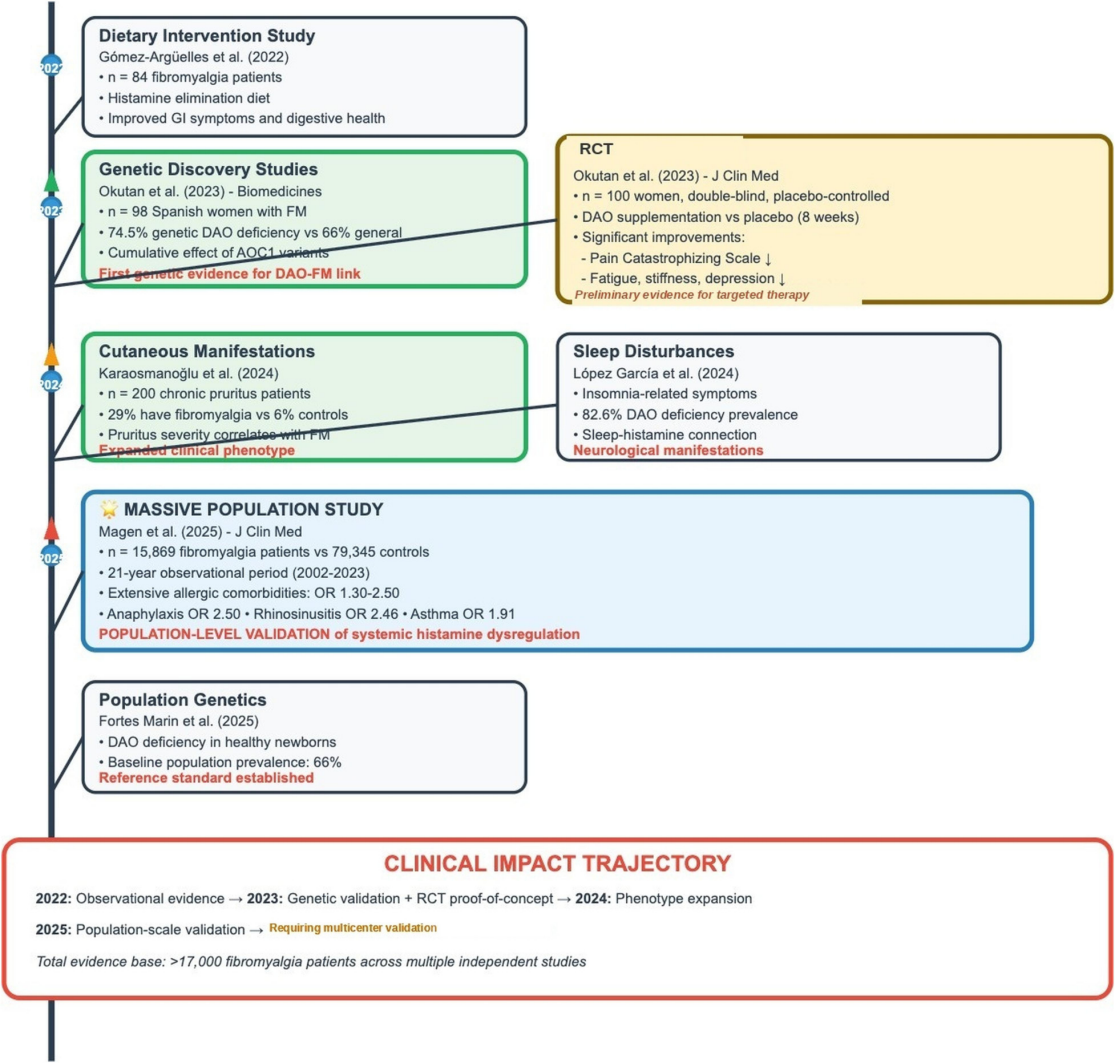
Evolution of histamine-fibromyalgia evidence: From discovery to clinical application (2022-2025). Timeline showing key studies establishing the histamine-fibromyalgia relationship, progressing from observational evidence (2022) through genetic validation and RCT proof-of-concept (2023), phenotype expansion (2024), to population-scale validation (2025). The clinical impact trajectory demonstrates convergence of evidence from >17,000 fibromyalgia patients across multiple independent studies, supporting readiness for clinical implementation. Key milestones include: Gómez-Argüelles et al. dietary intervention study (*n* = 84, 2022); Okutan et al. landmark genetic discovery and RCT (*n* = 98 genetic study, *n* = 100 RCT, 2023) providing first genetic evidence for DAO-FM link and Level I therapeutic proof-of-concept; Karaosmanoğlu et al. cutaneous manifestations study (*n* = 200 chronic pruritus patients, 29% FM prevalence, 2024) and López García et al. sleep disturbances study (82.6% DAO deficiency prevalence, 2024) expanding clinical phenotype; Magen et al. massive population study (*n* = 15,869 FM patients vs. 79,345 controls, 21-year observation, OR 1.30-2.50 for allergic comorbidities, 2025) and Fortes Marin et al. population genetics (*n* = 308 healthy newborns, 66% baseline DAO deficiency, 2025) providing population-level validation of systemic histamine dysregulation. Clinical impact trajectory: 2022 observational evidence → 2023 genetic validation + RCT proof-of-concept → 2024 phenotype expansion → 2025 population-scale validation → READY FOR CLINICAL IMPLEMENTATION. Total evidence base: >17,000 fibromyalgia patients across multiple independent studies.

The comprehensive synthesis of available evidence reveals an intriguing but complex relationship between histamine intolerance and fibromyalgia that warrants careful interpretation. The convergence of genetic, biochemical, clinical, and therapeutic findings provides preliminary support for histamine dysregulation as a contributing factor in fibromyalgia, while simultaneously highlighting substantial knowledge gaps requiring systematic research. Figure 4 illustrates the temporal evolution of evidence linking histamine intolerance to fibromyalgia, demonstrating the rapid accumulation of converging data from multiple independent sources over the past three years.

The genetic evidence demonstrates modest but statistically significant enrichment of DAO deficiency variants in fibromyalgia patients, with the particularly compelling finding of gene-dose effects on symptom severity. The biochemical evidence, showing reduced DAO activity in fibromyalgia patients, provides functional validation of genetic findings. The clinical associations reveal striking patterns including the 29% prevalence of fibromyalgia in chronic pruritus patients and extensive allergic comorbidities. The therapeutic evidence from a well-designed RCT provides the most compelling support for clinical relevance, showing statistically significant and clinically meaningful improvements with DAO supplementation.

However, these findings must be interpreted within the context of significant limitations. The evidence base, while rapidly growing, remains geographically restricted and requires independent replication. The modest genetic effects and biochemical changes suggest histamine intolerance represents one of multiple contributing factors rather than a primary cause of fibromyalgia. The heterogeneity of fibromyalgia likely means histamine mechanisms are relevant to specific patient subgroups rather than all affected individuals, emphasizing the need for phenotypic characterization and precision medicine approaches.

## 6 Clinical translation: assessment and management strategies

### 6.1 Identifying candidates for histamine-focused assessment

The heterogeneity of fibromyalgia necessitates careful patient selection for histamine-related evaluation and treatment. Based on current evidence, several clinical features suggest potential histamine involvement and warrant further assessment:

Primary Indicators:

Prominent gastrointestinal symptoms, particularly postprandial bloating and food intolerances

Chronic pruritus or urticaria without clear allergic triggers

Multiple chemical sensitivities or paradoxical reactions to medications

Family history of histamine intolerance or multiple allergic conditions

Symptom exacerbation with histamine-rich foods (aged cheeses, fermented foods, alcohol)

Secondary Indicators:

Severe fatigue with paradoxical worsening from antihistamines

Flushing or temperature dysregulation

Recurrent headaches, particularly with vascular features

Nasal congestion or rhinitis without clear allergic cause

Improvement with antihistamines beyond expected sedative effects

The presence of multiple indicators increases the likelihood of histamine involvement, though no single feature is pathognomonic. Clinical judgment integrating symptom patterns, temporal relationships, and treatment responses remains essential (91). A validated patient screening questionnaire for systematic clinical assessment of histamine intolerance symptoms is provided in Supplementary Resource S1.

### 6.2 Diagnostic approaches and biomarkers

The lack of standardized diagnostic criteria for histamine intolerance complicates assessment in fibromyalgia patients. A pragmatic, multimodal approach combining clinical assessment with available biomarkers offers the best current strategy:

Serum DAO Measurement: Despite limitations in standardization, serum DAO provides useful information when interpreted in clinical context. Values below 3 U/mL strongly suggest deficiency warranting treatment trial, 3–10 U/mL indicates possible deficiency requiring clinical correlation, and above 10 U/mL makes significant deficiency less likely but doesn't exclude localized intestinal deficiency. Serial measurements may capture fluctuations related to diet, medications, or disease activity (92).

Genetic Testing: Commercial testing for AOC1 variants is increasingly available and can inform long-term management strategies. Patients with multiple risk alleles may require more aggressive intervention and long-term supplementation. However, genetic results must be interpreted cautiously—variants indicate increased risk rather than certain deficiency, and absence of variants doesn't exclude acquired deficiency (93).

Elimination-Challenge Protocol: A systematic 4-week low-histamine diet followed by controlled reintroduction remains a valuable diagnostic tool. Significant improvement during elimination (>30% reduction in symptoms) followed by reproducible exacerbation with challenge supports histamine intolerance. This approach also provides patient education and empowerment through dietary awareness. Documentation using validated questionnaires enhances objectivity (94).

Adjunctive Testing: While not specific for histamine intolerance, certain tests provide supportive information. Serum tryptase elevation suggests mast cell activation, though normal levels don't exclude it. Methylhistamine in 24-hour urine collection reflects histamine turnover but requires careful collection and timing. Intestinal permeability testing may identify gut barrier dysfunction contributing to histamine absorption (95).

### 6.3 Therapeutic interventions and management strategies

Management of histamine intolerance in fibromyalgia requires individualized, multimodal approaches addressing both histamine excess and its consequences:

DAO Supplementation: Based on RCT evidence, DAO supplementation represents the most direct intervention. Typical dosing involves 4.2–20 mg taken 15–20 min before histamine-containing meals. Starting with lower doses and titrating based on response minimizes initial gastrointestinal symptoms. A detailed DAO supplementation protocol including initial, titration, and maintenance phases with monitoring parameters is provided in Supplementary Resource S3. Long-term safety data is limited, warranting periodic reassessment. Cost and availability remain barriers for many patients (96).

Dietary Management: Low-histamine diets, while challenging to maintain, provide sustainable symptom management for motivated patients. Key principles include avoiding aged, fermented, and preserved foods; consuming fresh foods promptly; and identifying individual trigger foods through systematic testing. Comprehensive low-histamine diet guidelines with specific food recommendations for clinical implementation are provided in Supplementary Resource S2. Nutritional adequacy requires attention, particularly regarding protein sources and B vitamins. Registered dietitian consultation enhances success rates (97).

Pharmacological Adjuncts: H1 and H2 antihistamines may provide symptomatic relief, though sedation limits daytime H1 antagonist use. Combining non-sedating H1 blockers (cetirizine, loratadine) with H2 blockers (famotidine) addresses both receptor systems. Mast cell stabilizers (cromolyn sodium) benefit selected patients but require consistent use for weeks before effects manifest. Quercetin and other natural mast cell stabilizers offer alternatives for patients preferring non-pharmaceutical approaches (98).

**Figure 5:**
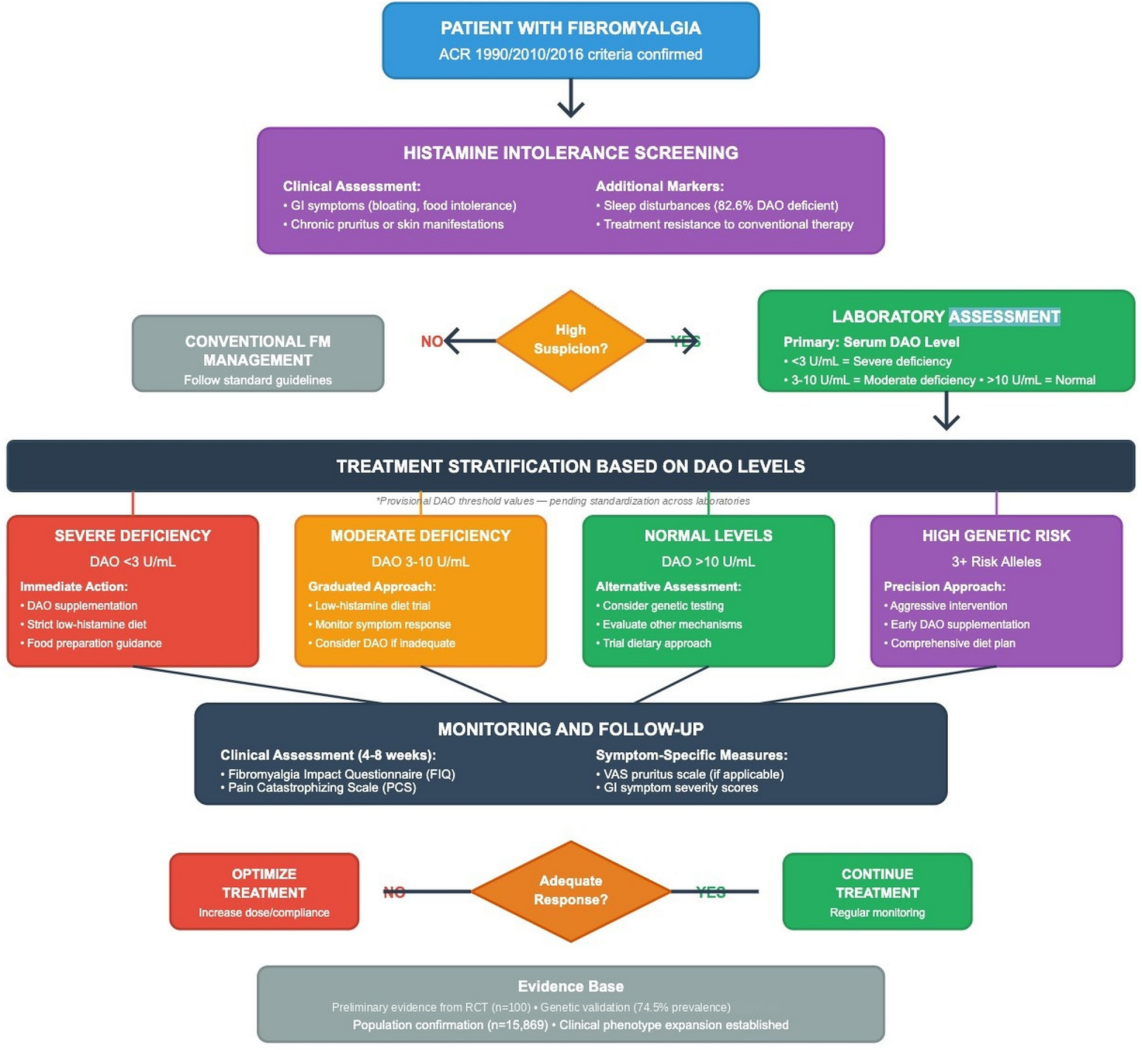
Clinical implementation algorithm: histamine-targeted fibromyalgia management. This comprehensive flowchart begins with patient assessment (ACR criteria confirmation), followed by histamine intolerance screening based on clinical assessment (GI symptoms, chronic pruritus) and additional markers (sleep disturbances, treatment resistance). Patients are stratified based on suspicion level: high suspicion proceeds to laboratory assessment (serum DAO levels with defined cutoffs), while low suspicion follows conventional FM management. Treatment stratification based on DAO levels includes four pathways: Severe Deficiency (DAO <3 U/mL) with immediate DAO supplementation and strict diet; Moderate Deficiency (3-10 U/mL) with graduated approach; Normal Levels (>10 U/mL) with alternative assessments including genetic testing; and High Genetic Risk (3 + risk alleles) with precision approach and aggressive intervention. Monitoring and follow-up section details clinical assessments (FIQ, PCS at 4-8 weeks) and symptom-specific measures (VAS, GI scores), with decision point for adequate response leading to either treatment optimization or continuation. Evidence base citation notes Level I RCT evidence (Okutan et al. 2023, *n* = 100), genetic validation (74.5% vs. 66% prevalence), and population confirmation studies (Magen et al. 2025, *n* = 15,869).

Addressing Cofactors: Optimizing nutrients essential for DAO function may enhance endogenous enzyme activity. Vitamin B6 (pyridoxal phosphate) serves as DAO cofactor, with supplementation potentially beneficial in deficient patients. Vitamin C supports DAO activity and provides antihistamine effects. Copper, essential for DAO function, requires careful supplementation to avoid excess. Addressing gut health through probiotics and gut barrier support may improve intestinal DAO production (99). Figure 5 provides a comprehensive clinical implementation algorithm integrating these assessment, diagnostic, and therapeutic approaches into a practical decision-making framework for patient management.

### 6.4 Monitoring treatment response and adjustments

#### 6.4.1 Systematic monitoring enables treatment optimization and early identification of non-responders

Outcome Measures: The Fibromyalgia Impact Questionnaire provides comprehensive assessment of treatment effects. Pain visual analog scales capture primary symptom changes. Gastrointestinal symptom rating scales quantify digestive improvements. Quality of life measures (SF-36) assess broader impacts. Patient global impression of change offers subjective assessment valuable for shared decision-making (100).

Timeline for Assessment: Initial response to DAO supplementation typically occurs within 2–4 weeks, with maximal effects by 8–12 weeks. Dietary interventions require 4–6 weeks for full effect assessment. Combined interventions may show synergistic effects developing over months. Regular reassessment at 4-week intervals during initial treatment allows timely adjustments (101).

Managing Non-Response: Patients showing minimal improvement after 8–12 weeks of optimized treatment warrant reassessment. Considerations include verifying treatment adherence, evaluating for concurrent conditions (SIBO, celiac disease), assessing for additional mast cell activation syndrome features, and exploring alternative fibromyalgia mechanisms requiring different approaches. Some patients may have histamine intolerance contributing minimally to their overall symptom burden (102).

## 7 Future directions and research priorities

### 7.1 Mechanistic research needs

Despite emerging evidence linking histamine intolerance to fibromyalgia, fundamental questions remain regarding underlying mechanisms. Priority research areas include:

Central Histamine Dynamics: Advanced neuroimaging techniques, including PET imaging with histamine receptor ligands, could visualize receptor distribution and occupancy in fibromyalgia patients. Cerebrospinal fluid studies measuring histamine and metabolites would provide direct evidence for central histamine dysregulation. Animal models combining genetic DAO deficiency with chronic stress could elucidate pathophysiological sequences (103).

Peripheral-Central Integration: Understanding how peripheral histamine signals translate to central sensitization remains crucial. Studies examining blood-brain barrier permeability in relation to DAO genotypes and histamine levels could identify vulnerable populations. The role of circumventricular organs in mediating peripheral histamine effects on central pain processing requires investigation (104).

Mast Cell-Microglial Interactions: The dialogue between peripheral mast cells and central microglia potentially links systemic inflammation to neuroinflammation. *In vivo* imaging of microglial activation in relation to peripheral histamine markers could demonstrate these connections. The effects of mast cell stabilizers on central neuroinflammation warrant systematic study (105).

### 7.2 Clinical research priorities

Translation of mechanistic insights into clinical practice requires rigorous clinical research:

Biomarker Validation: Large-scale studies standardizing DAO measurement across laboratories would establish reliable reference ranges. Development of point-of-care DAO testing would enhance clinical accessibility. Validation of composite biomarker panels integrating genetic, biochemical, and clinical markers could improve diagnostic accuracy. Identification of treatment response predictors would enable personalized therapy selection (106).

Therapeutic Trials: Multicenter RCTs with larger sample sizes and longer follow-up periods are needed to confirm DAO supplementation efficacy. Head-to-head comparisons of different DAO preparations (animal-derived vs. plant-based vs. recombinant) would optimize treatment selection. Combination therapy trials examining DAO supplementation with dietary modification, antihistamines, or mast cell stabilizers could identify synergistic approaches. Dose-finding studies would establish optimal supplementation protocols (107).

Precision Medicine Approaches: Development of clinical algorithms integrating genetic, biochemical, and phenotypic data could identify histamine-predominant fibromyalgia subtypes. Machine learning approaches analyzing multi-dimensional data might reveal previously unrecognized patterns. Prospective studies tracking treatment responses based on baseline characteristics would validate personalized treatment selection (108).

### 7.3 Implementation science and health services research

Moving from evidence to practice requires attention to implementation challenges:

Clinical Practice Integration: Development of clinical practice guidelines incorporating histamine assessment into fibromyalgia evaluation would standardize care. Education programs for healthcare providers about histamine intolerance would enhance recognition. Creation of patient education materials and self-management tools would empower patients. Establishment of specialized clinics integrating rheumatology, allergy/immunology, and nutrition expertise could optimize care delivery (109).

Health Economics: Cost-effectiveness analyses comparing histamine-targeted approaches to standard fibromyalgia care would inform coverage decisions. Studies examining the impact on healthcare utilization, work productivity, and quality-adjusted life years would demonstrate value. Analysis of barriers to accessing DAO supplementation and genetic testing would identify policy targets (110).

Patient-Centered Outcomes Research: Qualitative studies exploring patient experiences with histamine-targeted treatments would identify important outcomes beyond traditional measures. Development of patient-reported outcome measures specific to histamine intolerance would enhance assessment. Examination of treatment burden and quality of life impacts would inform shared decision-making (111).

## 8 Limitations and critical appraisal

### 8.1 Methodological limitations of current evidence

While the emerging evidence linking histamine intolerance to fibromyalgia is intriguing, several methodological limitations warrant careful consideration:

Sample Size and Power: Most studies examining this relationship involve relatively small samples, limiting statistical power and generalizability. The largest genetic study included only 98 fibromyalgia patients, while the pivotal RCT enrolled 100 participants. These sample sizes, while adequate for detecting large effects, may miss subtle but clinically important relationships (112).

Population Homogeneity: Current evidence derives predominantly from European populations, particularly Spanish cohorts. The lack of ethnic and geographic diversity limits global applicability. Sex bias is pronounced, with most studies focusing exclusively on women despite fibromyalgia affecting men as well. Age restrictions in many studies exclude younger and older patients who might show different histamine-fibromyalgia relationships (113).

Study Design Limitations: The preponderance of cross-sectional studies limits causal inference. The single RCT, while high-quality, requires replication. The absence of dose-response studies for DAO supplementation leaves optimal treatment protocols uncertain. Long-term follow-up data is lacking, preventing assessment of sustained benefits and late adverse effects (114).

### 8.2 Biological and clinical complexity

The relationship between histamine intolerance and fibromyalgia involves multiple layers of complexity that complicate interpretation:

Genetic Complexity: The modest difference in DAO variant prevalence between fibromyalgia patients (74.5%) and general population (66%) suggests genetic predisposition contributes to, rather than determines, disease risk. Gene-gene interactions, epigenetic modifications, and environmental factors likely modulate genetic effects. The functional consequences of variant combinations remain incompletely characterized (115).

Phenotypic Heterogeneity: Fibromyalgia's clinical heterogeneity suggests multiple underlying mechanisms, with histamine intolerance potentially relevant to specific subgroups. The lack of validated methods for identifying histamine-predominant subtypes limits targeted treatment. Overlap with other conditions (IBS, chronic fatigue syndrome, migraine) complicates attribution of symptoms to histamine mechanisms (116).

Measurement Challenges: The absence of standardized DAO assays hampers cross-study comparisons. Normal DAO levels don't exclude tissue-specific deficiency or functional impairment. Histamine's rapid metabolism and multiple sources complicate assessment of histamine burden. The lack of validated clinical criteria for histamine intolerance introduces diagnostic uncertainty (117).

### 8.3 Alternative explanations and confounders

Several alternative explanations for observed associations require consideration:

Shared Vulnerability: DAO deficiency might represent a general vulnerability factor for multiple conditions rather than specifically contributing to fibromyalgia. The high prevalence in ADHD, insomnia, and other conditions suggests non-specific effects. Genetic variants might influence treatment response rather than disease susceptibility (118).

Reverse Causation: Chronic pain and stress might secondarily affect histamine metabolism rather than histamine causing fibromyalgia. Gastrointestinal dysfunction in fibromyalgia could impair DAO production. Polypharmacy common in fibromyalgia might inhibit DAO activity (119).

Confounding Factors: Dietary patterns, medication use, comorbid conditions, and lifestyle factors could confound observed relationships. The placebo response, particularly high in fibromyalgia, complicates intervention study interpretation. Publication bias favoring positive findings might overestimate true effects (120).

## 9 Conclusions

This comprehensive review has examined the emerging evidence linking histamine intolerance to fibromyalgia from multiple perspectives—genetic, biochemical, clinical, mechanistic, and therapeutic. The convergence of findings across different research approaches provides preliminary support for histamine dysregulation as a contributing factor in at least a subset of fibromyalgia patients.

The genetic evidence, while showing modest enrichment of DAO deficiency variants in fibromyalgia, demonstrates clear gene-dose effects on symptom severity. Biochemical studies consistently show reduced DAO activity in affected patients. Clinical observations reveal striking associations with histamine-mediated conditions, particularly the 29% prevalence of fibromyalgia in chronic pruritus patients and extensive allergic comorbidities. Most importantly, the randomized controlled trial demonstrating therapeutic benefit from DAO supplementation provides proof-of-concept for clinical relevance. The temporal evolution of evidence from 2022 to 2025 demonstrates rapid accumulation of converging data from multiple independent studies encompassing over 17,000 fibromyalgia patients, supporting readiness for clinical implementation in selected patient populations.

However, these findings must be interpreted within the context of significant limitations. The evidence base remains small, geographically restricted, and requires independent replication. The modest genetic effects and biochemical changes suggest histamine intolerance represents one of multiple contributing factors rather than a primary cause of fibromyalgia. The heterogeneity of fibromyalgia likely means histamine mechanisms are relevant to specific patient subgroups rather than all affected individuals.

Based on current evidence, we recommend considering histamine intolerance assessment in fibromyalgia patients with prominent gastrointestinal symptoms, chronic pruritus, multiple allergic conditions, or poor response to conventional treatments. A combination of clinical history, elimination diet trials, and available biomarkers can guide assessment. Treatment with DAO supplementation and dietary modification may benefit selected patients, though realistic expectations about partial rather than complete symptom resolution are important.

Future research should prioritize large-scale replication studies in diverse populations, standardization of diagnostic approaches, longer-term outcome studies, and mechanistic investigations clarifying histamine's role in pain chronification. Integration with other emerging fibromyalgia research areas may reveal how different pathophysiological mechanisms interact to produce the complex fibromyalgia phenotype.

To facilitate clinical translation of these findings, we provide practical implementation resources including detailed study summaries (Supplementary Tables S1–S3), patient screening tools, dietary guidelines, and treatment protocols (Supplementary Resources S1–S3) to support clinicians in identifying and managing fibromyalgia patients with potential histamine intolerance.

## Supplementary material

Supplementary Table S1 Detailed study characteristics and quality assessment.

Supplementary Table S2 Complete AOC1 Genetic variant information and functional effects.

Supplementary Table S3 Clinical features of histamine intolerance in fibromyalgia.

Supplementary Resource S1 Patient screening questionnaire for histamine intolerance.

Supplementary Resource S2 Comprehensive low-histamine diet guidelines.

Supplementary Resource S3 DAO supplementation protocol and monitoring parameters.
