# Supplementary figures and images for: Is histamine intolerance a treatable subtype of fibromyalgia? evidence and clinical implications—narrative review
Source: Front Pain Res (Lausanne). 2026 Apr 30;7:1786437. doi: 10.3389/fpain.2026.1786437 (PMC13171840; doi:10.3389/fpain.2026.1786437)

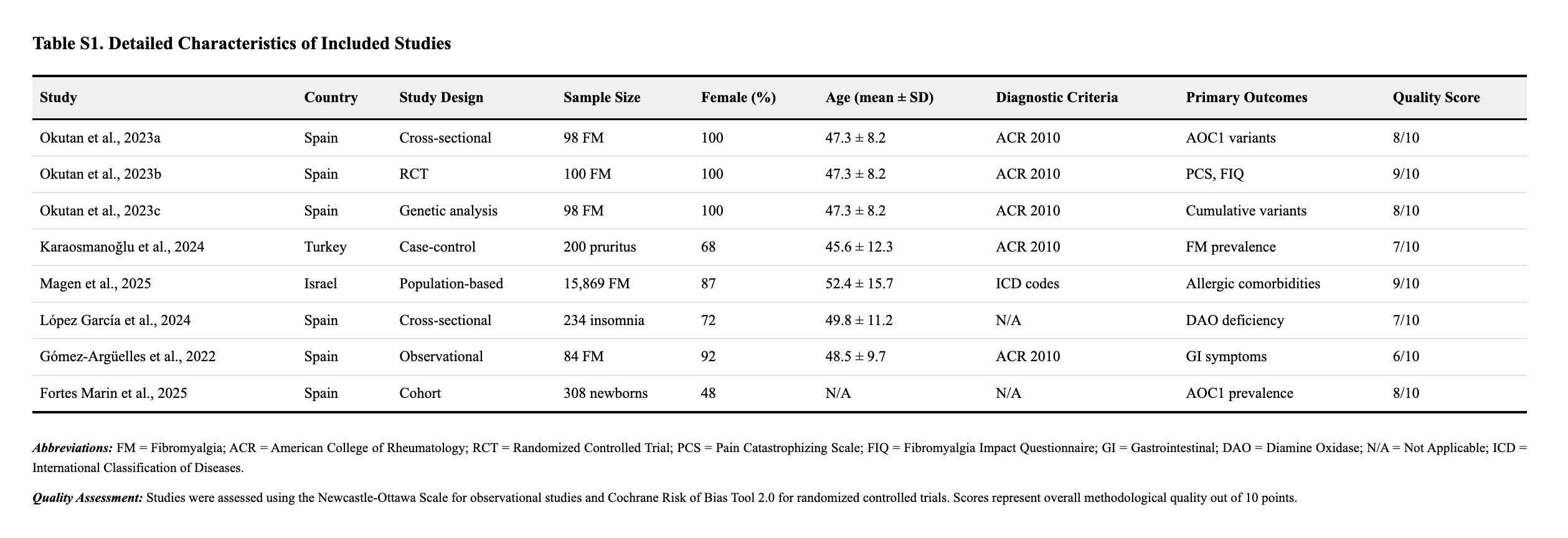

Supplement: Supplementary Table S1 — Detailed study characteristics and quality assessment. [file Image1.png]

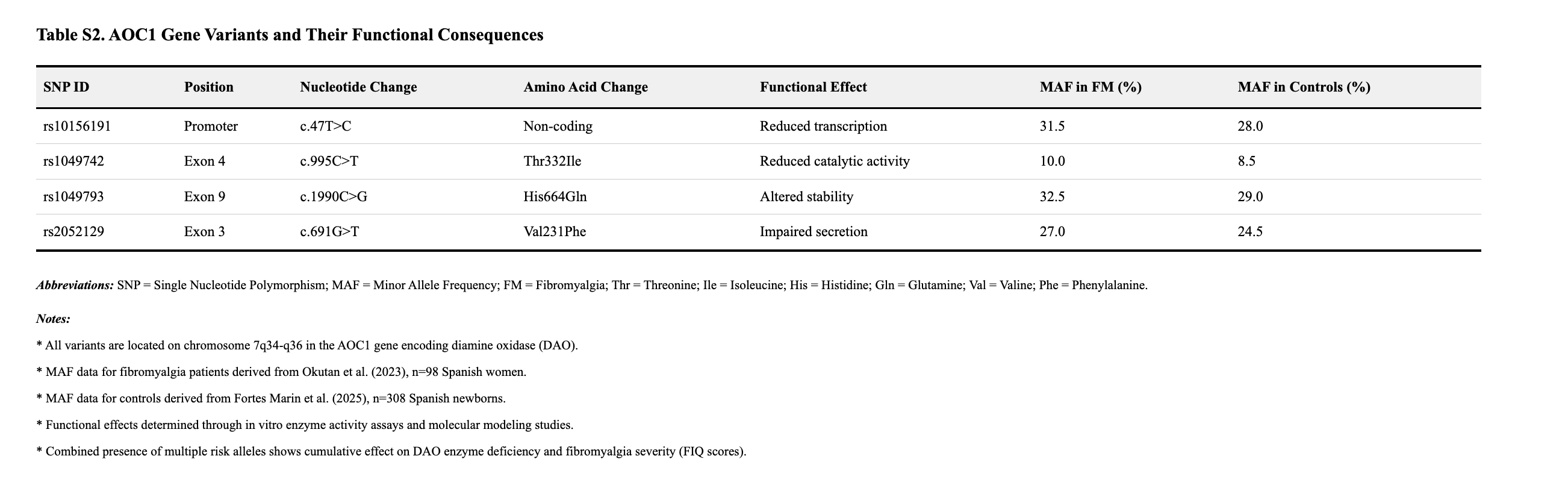

Supplement: Supplementary Table S2 — Complete AOC1 Genetic variant information and functional effects. [file Image2.png]

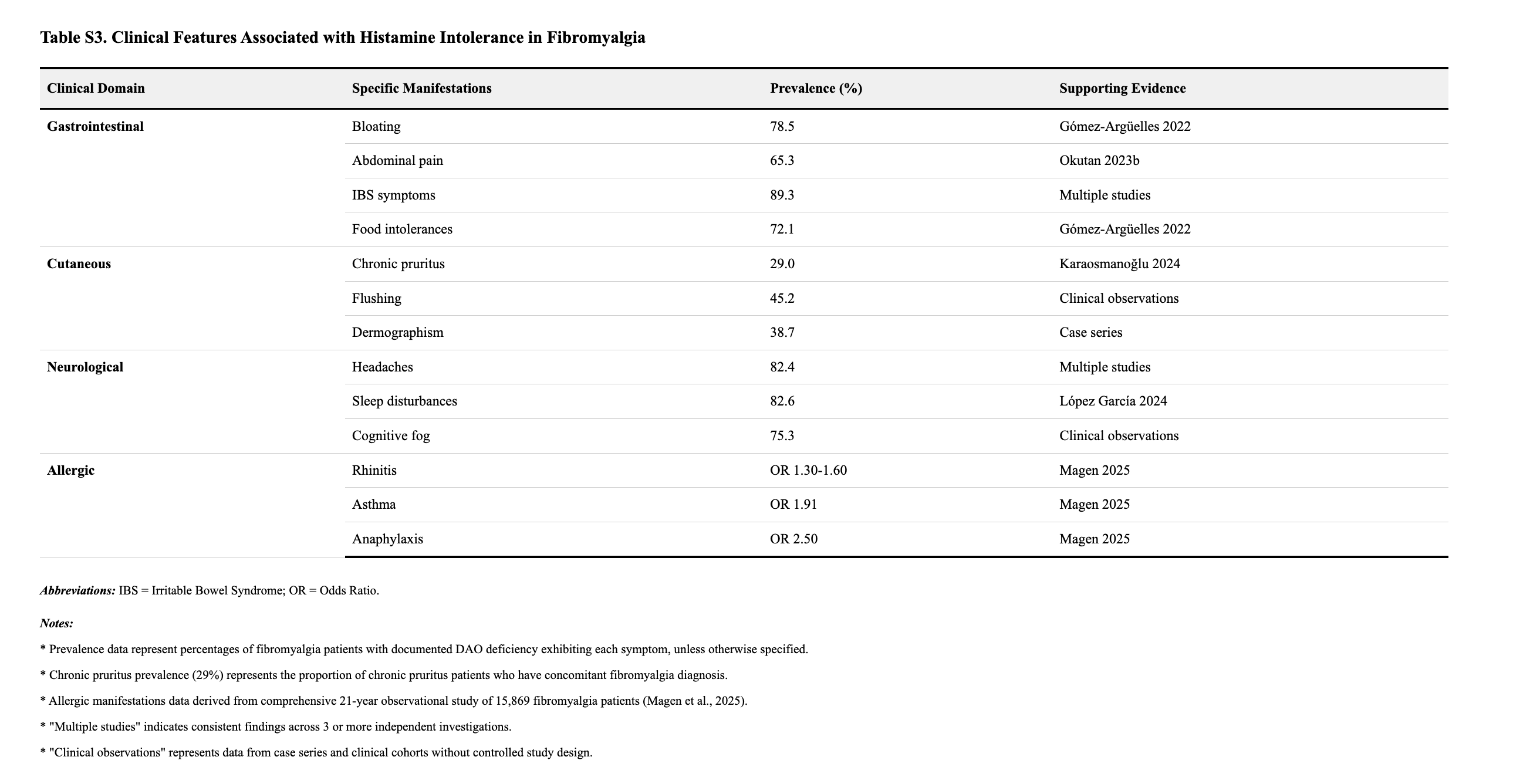

Supplement: Supplementary Table S3 — Clinical features of histamine intolerance in fibromyalgia. [file Image3.png]

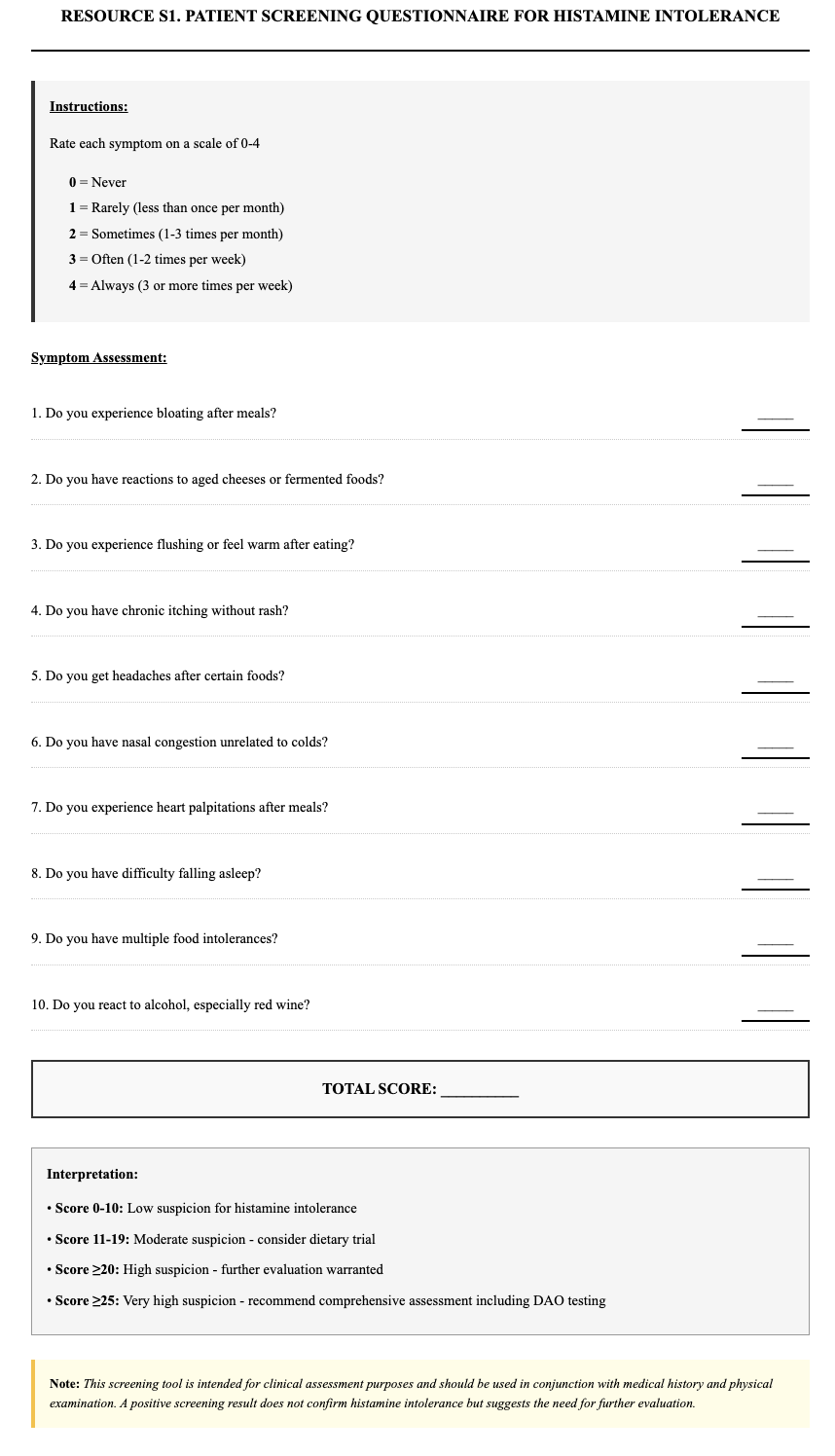

Supplement: Supplementary Resource S1 — Patient screening questionnaire for histamine intolerance. [file Image4.png]

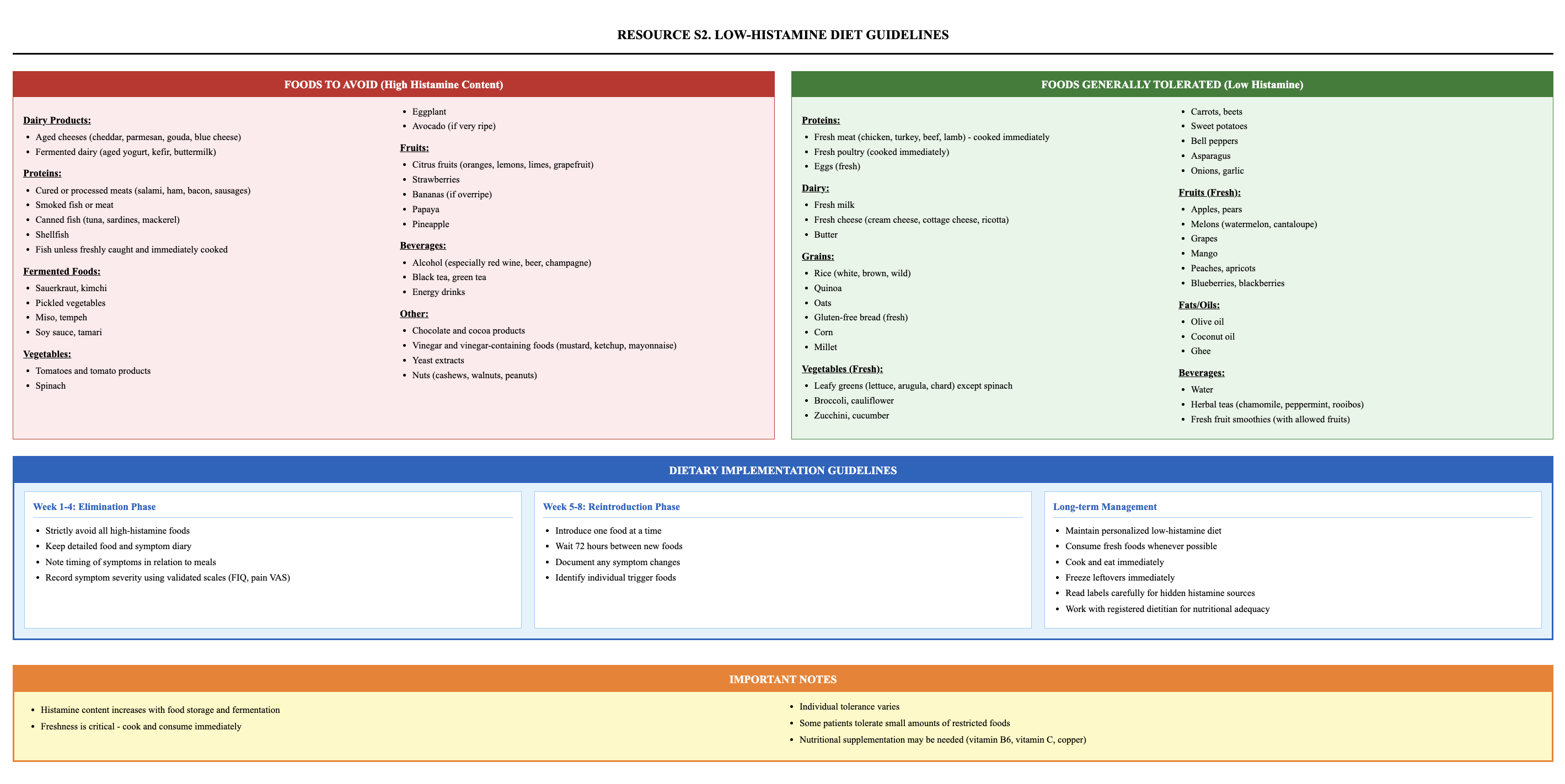

Supplement: Supplementary Resource S2 — Comprehensive low-histamine diet guidelines. [file Image5.png]

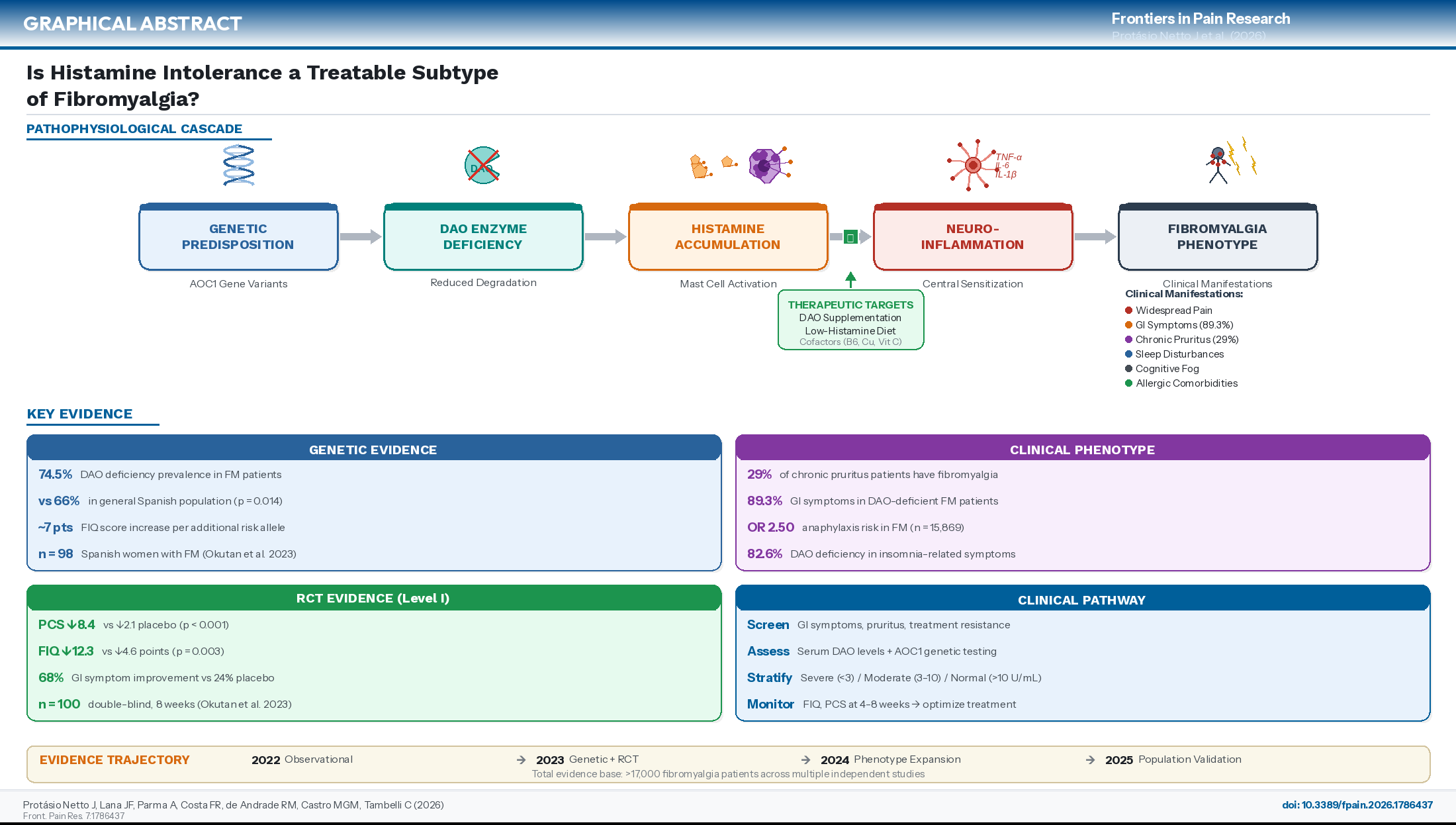

Supplement: Supplementary Resource S3 — DAO supplementation protocol and monitoring parameters. [file Image10.tiff]
