# Supplementary material for: Is histamine intolerance a treatable subtype of fibromyalgia? evidence and clinical implications—narrative review
Source: Front Pain Res (Lausanne). 2026 Apr 30;7:1786437. doi: 10.3389/fpain.2026.1786437 (PMC13171840; doi:10.3389/fpain.2026.1786437)
Supplement: Supplementary file 7 [file Datasheet1.pdf]

RESOURCE S3. DAO SUPPLEMENTATION PROTOCOL

INITIAL ASSESSMENT

Before Starting DAO Supplementation:

- ☐ Confirm fibromyalgia diagnosis (ACR 2010/2016 criteria)
- ☐ Complete histamine intolerance screening questionnaire
- ☐ Consider baseline DAO level measurement (if available)
- ☐ Document baseline symptoms using validated tools:
  - Fibromyalgia Impact Questionnaire (FIQ)
  - Pain Catastrophizing Scale (PCS)
  - Visual Analog Scale (VAS) for pain
  - Gastrointestinal symptom rating scale
- ☐ Review current medications for DAO inhibitors
- ☐ Screen for contraindications

ASSESSMENT SCHEDULE

Time Point	Assessments
**Baseline**	FIQ, PCS, Pain VAS, GI symptoms, Sleep quality, QoL (SF-36)
**Week 4**	Repeat all baseline measures, Assess tolerability, Review diary, Adjust dosing
**Week 8**	Complete reassessment, Determine response, Long-term strategy

SUPPLEMENTATION PROTOCOL

PHASE 1: Initial Phase (Weeks 1-2)

Dosing:
- 4.2 mg DAO enzyme
- Timing: 15-20 min before ONE main meal
- Start with meal likely to contain histamine

Monitoring:
- Daily symptom diary
- Note adverse effects
- Record meal timing and content

Expected Response:
- May notice initial GI adjustment
- Symptom improvement may begin

PHASE 2: Titration Phase (Weeks 3-4)

Dosing:
- Increase to 4.2 mg before TWO main meals
- Maintain 15-20 min pre-meal timing
- Consider adding before breakfast if needed

Monitoring:
- Continue daily symptom diary
- Assess symptom patterns
- Evaluate tolerance to increased dose

Expected Response:
- Progressive symptom improvement
- Better tolerance to histamine foods
- Reduction in postprandial symptoms

PHASE 3: Maintenance Phase (Weeks 5-8)

Dosing:
- 4.2-8.4 mg before EACH histamine meal
- Individualize based on response
- Maximum daily dose: 20 mg
- Continue pre-meal timing (15-20 min)

Monitoring:
- Weekly symptom assessment
- Track dietary compliance
- Document response patterns
- Identify optimal dosing schedule

Expected Response:
- Maximal therapeutic benefit
- Stabilized symptom control
- Improved quality of life measures

RESPONSE CRITERIA

Treatment Success:
- ≥30% reduction in FIQ score
- ≥2-point reduction in pain VAS
- Subjective improvement in GI symptoms
- Patient global impression: "much improved"

Partial Response:
- 15-29% reduction in FIQ score
- Some symptom improvement
- Consider dose optimization
- Consider combination therapy

Non-Response:
- <15% improvement after 8 weeks
- Consider alternative diagnoses
- Reassess histamine intolerance
- Evaluate other mechanisms

ADVERSE EFFECTS & LONG-TERM MANAGEMENT

Common Adverse Effects (mild, transient):
- Mild gastrointestinal discomfort (12%)
- Bloating or gas
- Nausea (rare)

Management:
- Reduce dose temporarily
- Take with small amount of food if needed
- Usually resolves within 1-2 weeks
- Consider alternative DAO preparation

Discontinuation Criteria:
- Persistent severe GI symptoms
- Allergic reaction
- No improvement after 12 weeks optimized therapy

For Responders:
- Continue effective dose
- Maintain low-histamine diet as adjunct
- Periodic reassessment (every 3-6 months)
- Monitor for sustained benefit
- Consider periodic "treatment holidays"

Combination Strategies:
- DAO supplementation + low-histamine diet
- Consider H1/H2 antihistamines if needed
- Mast cell stabilizers in selected cases
- Address cofactor deficiencies (B6, vitamin C, copper)

SPECIAL CONSIDERATIONS

Pregnancy/Lactation:
- Limited safety data
- Consult obstetrician
- Consider risks vs benefits

Drug Interactions:
- DAO inhibitors: NSAIDs, some antibiotics, certain antidepressants
- Separate DAO from meds by 2+ hours

Product Selection:
- Standardized DAO content
- Third-party tested
- GMP-certified
- Clear enzyme activity labeling

Cost Considerations:
- Discuss cost-effectiveness
- Insurance coverage varies
- Consider trial period first

CLINICAL DECISION POINTS

✓ When to Continue:
- Clear symptom improvement
- Good tolerability
- Objective measure improvements
- Patient satisfaction

⚠ When to Modify:
- Partial response - adjust dose
- Inconsistent response - evaluate adherence
- Plateau - add complementary strategies

✗ When to Discontinue:
- No response after 12 weeks
- Unacceptable adverse effects
- Patient preference
- Alternative explanation found

DOCUMENTATION TEMPLATE

Patient Name: ______________ **Date:** ______________

Baseline Assessment (Date: __________)
- ☐ FIQ Score: ________
- ☐ PCS Score: ________
- ☐ Pain VAS: ________
- ☐ GI Symptom Score: ________
- ☐ DAO Level: ________ U/mL

Week 4 Assessment (Date: __________)
- ☐ FIQ Score: ________ (Change: ______%)
- ☐ PCS Score: ________ (Change: ______%)
- ☐ Pain VAS: ________ (Change: ______)
- ☐ Adverse Effects: Yes ☐ No ☐
- ☐ Dosing Adjustment: ______________

Week 8 Assessment (Date: __________)
- ☐ FIQ Score: ________ (Change: ______%)
- ☐ PCS Score: ________ (Change: ______%)
- ☐ Pain VAS: ________ (Change: ______)
- ☐ Patient Global Impression: ______________
- ☐ Treatment Decision: Continue ☐ Modify ☐ Discontinue ☐

IMPORTANT DISCLAIMER: *These clinical resources are intended to support healthcare providers in the assessment and management of histamine intolerance in fibromyalgia patients. They should be used as part of comprehensive clinical evaluation and not as standalone diagnostic or treatment tools. Individual patient factors, comorbidities, and clinical judgment should guide all management decisions. The evidence base for histamine intolerance in fibromyalgia is still emerging, and treatment should be individualized based on patient response and preferences.*
